# Structural, functional and molecular analysis of the effects of aging in the small intestine and colon of C57BL/6J mice

**DOI:** 10.1186/1755-8794-5-38

**Published:** 2012-08-28

**Authors:** Wilma T Steegenga, Nicole JW de Wit, Mark V Boekschoten, Noortje IJssennagger, Carolien Lute, Shohreh Keshtkar, Mechteld M Grootte Bromhaar, Ellen Kampman, Lisette C de Groot, Michael Muller

**Affiliations:** 1 Division of Human Nutrition, Wageningen University, Wageningen, The Netherlands; 2 The Netherlands Nutrigenomics Centre, TI Food and Nutrition, Wageningen, The Netherlands

**Keywords:** Aging, Small intestine, Colon, Transcriptomics, Fat, Diet, DNA methylation, C57BL/6J mice, Morphology, Microbiota

## Abstract

**Background:**

By regulating digestion and absorption of nutrients and providing a barrier against the external environment the intestine provides a crucial contribution to the maintenance of health. To what extent aging-related changes in the intestinal system contribute to the functional decline associated with aging is still under debate.

**Methods:**

Young (4 M) and old (21 M) male C57BL/6J mice were fed a control low-fat (10E%) or a high-fat diet (45E%) for 2 weeks. During the intervention gross energy intake and energy excretion in the feces were measured. After sacrifice the small and large intestine were isolated and the small intestine was divided in three equal parts. Swiss rolls were prepared of each of the isolated segments for histological analysis and the luminal content was isolated to examine alterations in the microflora with 16S rRNA Q-PCR. Furthermore, mucosal scrapings were isolated from each segment to determine differential gene expression by microarray analysis and global DNA methylation by pyrosequencing.

**Results:**

Digestible energy intake was similar between the two age groups on both the control and the high-fat diet. Microarray analysis on RNA from intestinal scrapings showed no marked changes in expression of genes involved in metabolic processes. Decreased expression of Cubilin was observed in the intestine of 21-month-old mice, which might contribute to aging-induced vitamin B12 deficiency. Furthermore, microarray data analysis revealed enhanced expression of a large number of genes involved in immune response and inflammation in the colon, but not in the small intestine of the 21-month-old mice. Aging-induced global hypomethylation was observed in the colon and the distal part of the small intestine, but not in the first two sections of the small intestine.

**Conclusion:**

In 21-month old mice the most pronounced effects of aging were observed in the colon, whereas very few changes were observed in the small intestine.

## Background

The most well established functions of the small intestine are the digestion of food and absorption of nutrients. The main functions of the colon are the absorption of water and electrolytes and provide a reservoir for the fermentation of undigested macronutrients. To protect the body against detrimental invaders from the outside world, the intestine is armed with a complex and robust immune system [1,2]. The colon is also host to a broad variety of microorganisms that not only are responsible for the fermentation of undigested nutrients and the synthesis of essential vitamins, but also play a crucial role in the establishment of a proper immune system in postnatal life [1]. Recently, we have shown that physiological adaptations in the small intestine to dietary challenges may play a causal role in the development of diseases such as obesity and/or insulin resistance [3].

To enable these exclusive functions, the intestine has a unique and highly specialized tissue structure. A high efficiency of digestion and absorption is made possible by an enormous surface area created by intestinal folds, villi, microvilli, crypts and the glycocalyx in the small intestinal lumen. This large surface area facilitates optimal contact between food and digestive enzymes and creates a huge capacity for active and passive up-take of nutrients. In contrast to the small intestine, in the colon the increase in the surface area is realized by microvilli and crypts. The enormous contact area at the same time increases the risk of infections by either pathogens entering the body via the diet or by enteric commensal bacteria colonizing the intestinal lumen. Protection against invading microorganisms is provided by several defense mechanisms. Besides a mucous layer present on top of a continues layer of epithelial cells covering the surface of the intestine, a wide variety of immunological cells and tissue structures are present in the mucosa and submucosa of the intestinal wall [1,4,5].

Like all organs, the gastrointestinal tract does not escape the adverse effects of aging and various anomalies related to the digestive system occur with increasing frequency in our rapidly aging Western society. Impaired taste sensation, problems with swallowing, slower gastric emptying and intestinal and colonic transit, and altered levels of satiety hormones have been reported in the elderly [6-10]. To what extent impaired functioning of the small and large intestine contributes to the undernourished state often observed in the elderly is currently under debate. Aging-induced morphological [11-13] as well as functional changes in the intestine have been reported [8,14,15]. However, since these effects are often very subtle and the intestinal system has a highly adaptive and extensive reserve capacity [14], it is still questionable whether these changes play a role in aging-related malnutrition. Furthermore, it has been shown that aging can cause bacterial overgrowth in the small intestine [16,17] and promote changes in microbial composition in the colon [18-20]. In addition, reported age-related changes in DNA methylation of the mouse intestine [21] might play a role in the altered gene expression levels observed in the duodenum and colon of aging mice [22]. Together these observations demonstrate that although certain aspects of the aging intestine have been studied in a variety of independent studies, a comprehensive overview of the different features of the aging intestine is still lacking. Such a comprehensive study may prove essential to our understanding of how intestinal malfunction contributes to the undernourished condition often observed in the elderly.

In this study, we have carried out a detailed comparison of functional, structural and molecular features of the small intestine and the colon of 4- and 21-month-old male C57BL/6J mice. Mice of both age groups were exposed for two weeks to a low-fat or a high-fat diet to analyze the adaptive capacity of the intestine. During the intervention period body weight and the efficiency of energy up-take were measured. After sacrifice the small intestine and colon were isolated to analyze tissue morphology, luminal bacterial content and mucosal gene expression and DNA methylation. The results obtained in this study imply that at the age of 21 months, the intestine of C57BL/6J mice is functionally and structurally remarkably comparable to that of their 4-month-old counterparts. On a molecular level the main age-related changes in 21-month-old mice were found in the third part of the small intestine, and in particular in the colon.

## Methods

### Ethics statement

The institutional and national guidelines for the care and use of animals were followed and the experiment was approved by the Local Committee for Care and Use of Laboratory Animals at Wageningen University (code number: drs-2010048b).

### Animals and diets

Male C57BL/6J mice (age 2,5 and 19 months) were purchased from Janvier (Cedex, France) and were housed individually in the light and temperature-controlled animal facility (12/12 (light/dark), 20°C) of Wageningen University. The mice had free access to water and received standard laboratory chow (RMH-B, Arie Blok BV, Woerden, the Netherlands) for 12 days, followed by a run-in period for 3 weeks during which all mice received the low-fat diet (10E%) to adapt to the purified diets. At the start of the diet intervention, the young mice were divided into two groups of 6 mice and the old mice into two groups of 8 mice. A larger number of old mice were used to compensate for potential loss due to aging but none of the mice died during the experiment. Of each age group, half of the mice received a low-fat diet while the other half of the mice received a high-fat purified diet, as described in our previous study [3]. Both diets were based on Research Diets’ formulas D12450B/D12451, with adaptations regarding type of fat (palm oil instead of lard) and carbohydrates (Research diet services, Wijk bij Duurstede, The Netherlands). Thus, the high-fat diet mimics the ratio of saturated to monounsaturated to polyunsaturated fatty acids (40:40:20) in an average Western style human diet. The energy density of all nutrients except fat and starch was equal. After 2 weeks of diet intervention all mice were anaesthetized with a mixture of isofluorane (1.5%), nitrous oxide (70%) and oxygen (30%) in the postprandial state. From all sacrificed mice the small intestine and the colon were excised. The small intestine was divided in three equal parts, cut open longitudinally and shaken thoroughly in an eppendorf tube containing 1 ml PBS to isolate the luminal content. The luminal content of the colon was isolated removing the fecal parts with a spatula. After washing the proximal, middle and distal part of the small intestine and the colon in PBS Swiss rolls were prepared of the middle part of each segment for histomorphological analysis. For mRNA expression analysis and global DNA methylation analysis scrapings were made of the mucosa and submucosa of the remaining part of each section, snap frozen in liquid nitrogen and stored at -80°C. Scrapings were crushed on liquid nitrogen and divided into two portions, one for RNA and the other for DNA isolation.

### Energy intake and excretion

Food intake of each individual mouse was recorded during the run-in and the diet intervention period. Feces were collected from individual mice over a period of 24 hours and freeze dried after which the total amount of feces per mouse was measured. Energy content of the feces was determined with a bomb calorimeter (Isothermal Bomb Calorimeter, Cal2K, South Africa). The efficiency of energy intake was calculated by the formula: ((total energy intake – total energy exertion)/total energy intake)*100%.

### RNA isolation

Total RNA was isolated using TRIzol reagent (Invitrogen Breda, The Netherlands) according to the manufacturer’s instructions. The RNA was treated with DNAse and purified on columns using the RNAeasy microkit (Qiagen, Venlo, the Netherlands). RNA concentration was measured on a NanonDrop ND-1000 UV–vis spectrophotometer (Isogen, Maarsen, The Netherlands) and RNA integrity was checked on an Agilent 2100 Bioanalyzer (Agilent Technologies, Amsterdam, The Netherlands) with 6000 Nano Chips according to the manufacturer’s instructions. RNA was judged as suitable only if samples showed intact bands of 18S and 28S ribosomal RNA subunits, displayed no chromosomal peaks or RNA degradation products, and had a RNA integrity number (RIN) above 8.0.

### Microarray hybridization and analysis

Equal amounts of total RNA of all six young mice per diet group were pooled for the proximal, middle and distal part of the small intestine respectively and for the colon. For the old mice the same procedure was applied, but now all eight mice per diet group were taken. One hundred nanogram of pooled RNA was used for Whole Transcript cDNA synthesis (Affymetrix, Santa Clara, CA). Hybridization, washing and scanning of Affymetrix GeneChip Mouse Gene 1.0 ST arrays were carried out according to standard Affymetrix protocols. All arrays of the small intestine were hybridized in one experiment, and all colon samples were hybridized together in a second microarray experiment. Data analysis of the small intestine and colon was done separately. Arrays were normalized using the Robust Multi-array Average method [23,24]. Probe sets were defined according to Dai et al. [25]. In this method probes are assigned to unique gene identifiers, in this case Entrez IDs. The probes on the Gene 1.0 ST arrays represent 20,985 Entrez IDs. For the analysis only genes were taken into account that had an intensity value of > 50 on at least one array. Pancreas-specific genes (http://biogps.org) were removed from the analysis in order to omit an effect of potential pancreatic contamination. Changes in gene expression were calculated as fold change between young and old mice or between a high-fat and low-fat diet. For further analysis we used a fold change cut-off of > 2.0 to minimalize the inclusion of false positive differentially expressed genes. Array data have been submitted to the Gene Expression Omnibus, accession number GSE39975.

### cDNA synthesis and real-time quantitative PCR

To validate the microarray data and to check for inter-animal variation, RNA’s from individual mice were used. For each individual sample, single-stranded complementary DNA (cDNA) was synthesized from 1 μg of total RNA using the Reverse transcription system (iScript, Biorad) following the supplier's protocol. cDNA was PCR amplified with Platinum Taq DNA polymerase (all reagents were from Invitrogen). Primer sequences were retrieved from the online PrimerBank database [26], or otherwise designed using the Primer3 program [27] and the sequences of the primers used are listed in Table 1.

**Table 1:** Sequences of the primers used for Q-PCR analysis

**Gene**	**Sequence forward primer**	**Sequence reverse primer**
INFγ	ATGAACGCTACACACTGCATC	CCATCCTTTTGCCAGTTCCTC
IF44	AACTGACTGCTCGCAATAATGT	GTAACACAGCAATGCCTCTTGT
IF47	AGCAGATGAATCCGCTGATGT	CGTGGAAATTGGGTGTCCC
TNFα	CAACCTCCTCTCTGCCGTCAA	CGTGGAAATTGGGTGTCCC
IL1β	TGGTGTGTGACGTTCCCATT	CAGCACGAGGCTTTTTGTTG
IL6	CTTCCATCCAGTTGCCTTCTTG	AATTAAGCCTCCGACTTGTGAAG
Cubn	GAATGTGGCTCCAAGTCCCAT	ACGGCTAATGAAGGATGCAGA
36B4	AGCGCGTCCTGGCATTGTGTGG	GGGCAGCAGTGGTGGCAGCAGC

Primers were tested for specificity by BLAST analysis. Real-time quantitative PCR (Q-PCR) was performed using SYBR green and a MyIQ thermal cycler (Bio-Rad laboratories BV, Veenendaal, The Netherlands). The following thermal cycling conditions were used: 2 min at 94°C, followed by 40 cycles of 94°C for 15 s and 60°C for 45 s. PCR reactions to validate aging-induced differential gene expression were performed in duplicate and all samples were normalized to 36B4 expression.

### Histochemistry

Four-micrometer sections of paraffin-embedded parts of the proximal, middle and distal segment of the small intestine and of the colon were mounted on Superfrost microscope slides. These sections were dewaxed in xylene and rehydrated in a series of graded alcohols. After staining with Meyer's hematoxylin/eosine sections were mounted with DePex mounting medium (Gurr, BDH, Poole, Dorset, UK).

### Bacterial DNA extraction

DNA was extracted from the freeze-dried luminal content of the 4 sections of the intestine using the method described by Salonen *et al.*[28]. In short, approximately 0.1 g was used for mechanical and chemical lysis using 0.5 ml buffer (500 mM NaCl, 50 mM Tris–HCl (pH 8), 50 mM EDTA, 4% SDS) and 0.25 g of 0.1 mm zirconia beads and 3 mm glass beads. Nucleic acids were precipitated by addition of 130 μl, 10 M ammonium acetate, using one volume of isopropanol. Subsequently, DNA pellets were washed with 70% ethanol. Further purification of DNA was performed using the QiaAmp DNA Mini Stool Kit (Qiagen, Hilden, Germany). Finally, DNA was dissolved in 200 μl Tris/EDTA buffer and its purity and quantity were checked spectrophotometrically (ND-1000, nanoDrop technologies, Wilmington, USA).

### 16S rRNA Q-PCR

To quantify total bacteria using 16 S rRNA-specific primers Q-PCR analysis was in carried out as described above with a few modifications. The used primer sequence used were: 5’-ACTCCTACGGGAGGCAGCAG-3’ and 5’-ATTACCGCGGCTGCTGG-3’ The Q-PCR conditions used were: 95°C for 10 min, followed by 35 cycles of denaturation at 95°C for 15 sec, annealing temperature of 60°C for 20 sec, extension at 72°C for 30 sec and a final extension step at 72°C for 5 min.

### DNA isolation from scrapings of the small intestine and the colon

Genomic DNA was isolated from the crushed scraping by using DNeasy® Blood and Tissue Kit (Qiagen, Venlo, the Netherlands) according to the manufacturer’s instructions. The DNA was treated with RNase and eluted in Tris/EDTA buffer (pH 9.0). DNA purity and quantity were checked spectrophotometricaly (ND-1000, nanoDrop technologies, Wilmington, USA).

### DNA methylation analysis

700 ng of genomic DNA for each sample was bisulfite-treated using EZ-96 DNA Methylation-Gold™ Kit (Zymo Research, USA) and eluted in 16 μL of M-Elution Buffer. Included in the bisulfite-treatment was a separate methylation curve consisting of a mix of low and highly methylated mouse genomic DNA (0%, 25%, 50%, 75% and 100% of highly methylated DNA (EpigenDx, Worcester, USA)). The B1-element assay was performed in a 45 μL PCR reaction containing 6 μL of bisulfite-treated genomic DNA, PyroMark PCR Master Mix, CoralLoad Concentrate according to manufacturer’s instructions and 0.4 μM of each primer (Qiagen, Venlo, the Netherlands). The following thermal cycling conditions were used: 15 min at 95°C, followed by 45 cycles of 94°C for 30 s, 54°C for 30s, and 72°C for 40s, followed by a final elongation step at 72°C for 10 min. The primer sequence used for forward and biotinylated reverse primer were 5’-TTGGTTATTTTGGAATTTATTTTGTAGAT-3’ and 5’-biotin-TAATAACACACACCTTTAATCCCAACA-3’ respectively. B1-element methylation analysis was performed using Pyromark™ pyrosequencing technology (Biotage AB, Uppsala, Sweden). The PCR product was purified and made single-stranded using the Pyrosequencing Vacuum Prep Tool according to the manufacturer’s instructions (Qiagen, Venlo, The Netherlands). Sequencing primer (5’-TTTGGAATTTATTTTGTAGATTAG-3’) was annealed to the purified single-stranded PCR product and pyrosequencing was performed using the Q24 Pyrosequencing System (Qiagen, Venlo, The Netherlands) and CpG methylation was analyzed with the provided software.

### Statistical analysis

Physiological data are reported as the mean ± the standard error (SE). The differences between the mean values were tested for statistical significance by a one-way ANOVA. P-values < 0.05 are considered statistically significant.

## Results

### A high-fat diet, but not aging, affects intestinal function and morphology

**Figure 1:**
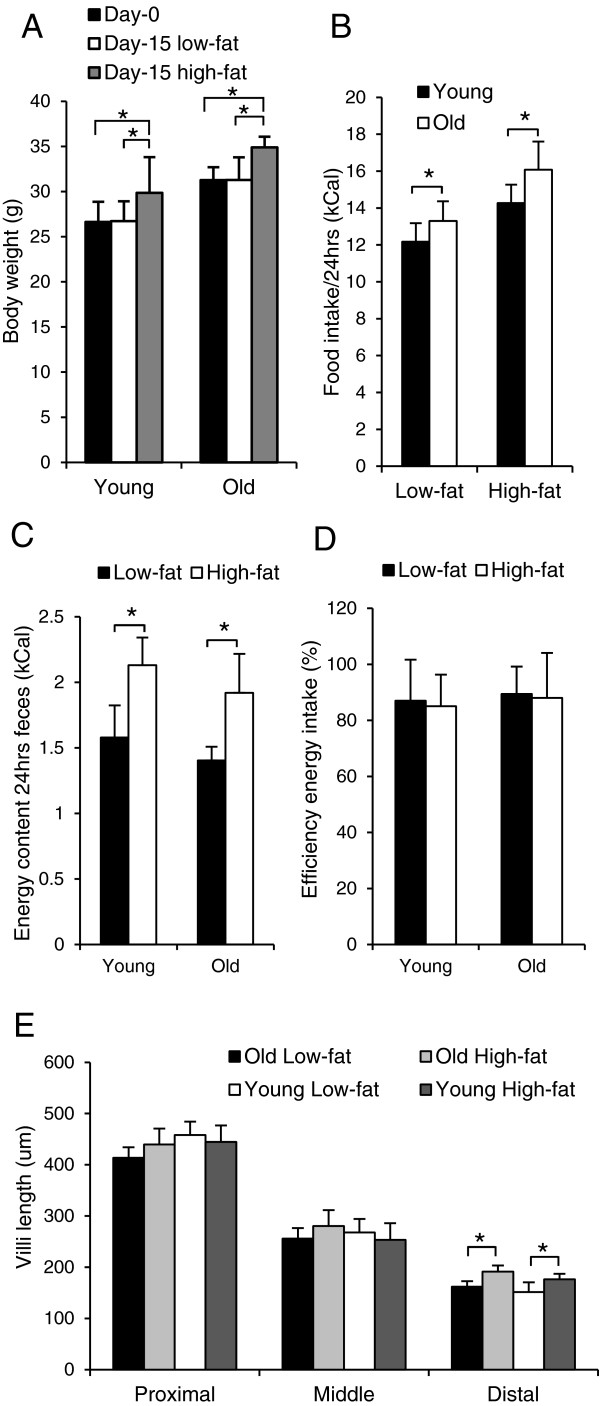
**Bodyweight development, efficiency of energy intake and small intestinal tissue morphology in young and old mice.** 4 and 21-month-old mice were exposed to a low-fat or a high-fat diet for 2 weeks. (**A**) Bodyweight, (**B**) food intake and (**C**) fecal energy excretion were measured. (**D**) The efficiency of energy up-take was calculated and (**E**) villi length of the proximal, middle and distal part of the intestine in H&E stained paraffin sections was compared between young and old mice. In all figure the mean values ± SE were presented, young/low-fat n = 6, young/high-fat n = 6, old/low-fat n = 8, old/high-fat n = 8, *p < 0.05.

To analyze changes in the ability of the intestine of aging mice to respond to a high-fat challenge, young and old male C57BL/6J mice were exposed to a control low-fat (10E%) or to a high-fat diet (45E%) and sacrificed at the age of 4 and 21 months. The specific diets were provided for two weeks since the results of our previous study showed a clear response in young mice fed a high-fat diet for this time period [3]. Although body weight of the young mice was lower than that of the old mice, both age groups showed a similar and significant weight gain after 2 weeks exposure to a high-fat diet (Figure 1A). Both young and old mice consumed significantly more energy (E)% on the high-fat diet than on the low-fat diet (Figure 1B), but no significant difference was found between the two age groups. In addition, remnant energy in feces from mice that received the high-fat diet was significantly higher than from mice fed the low-fat diet. This effect was also similar in young and old mice (Figure 1C). Based on gross energy intake and fecal out-put, we calculated that digestible energy uptake was equally efficient in young and old mice (Figure 1D). Taken together, these results indicate that the intestine of 21-month-old C57BL/6J mice is still fully capable in absorbing energy from the diet under both a low-fat condition and in response to a high-fat dietary challenge.

To perform its unique functions, the intestine requires a highly specialized tissue structure. Morphometric analysis of H&E stained sections of the proximal, middle and distal part of the small intestine showed, as expected, a marked decrease in villi length in the successive segments of the small intestine. However, again no significant differences between old and young mice were observed (Figure 1E). In the distal part of the small intestine a small but significant increase in villi length was observed after 2 weeks exposure to a high-fat diet in both old and young mice. No difference in crypt depth and villi width was found between old and young mice in all three parts of the small intestine (data not shown). In the colon, no differences between young and old mice in crypt depth on H&E stained sections or in the number of proliferating cells as measured by Ki67 staining were observed (data not shown). Thus, no changes were found in the functional capacity as well as in the tissue morphology of the intestine between 4- and 21- month-old mice.

### Aging-induced changes in gene expression are more pronounced in the colon than in the small intestine

**Figure 2:**
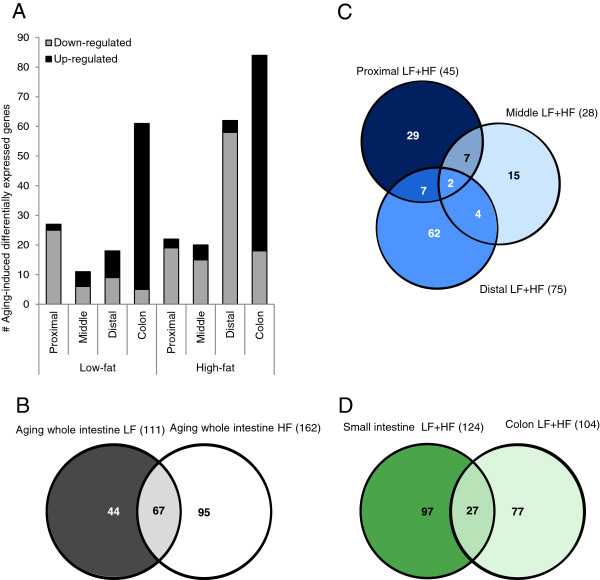
**Aging-induced differential gene expression in the 3 segments of the small intestine and the colon.** (**A**) Differences in basal gene expression between 4- and 21-month-old mice were calculated for each of the 4 segments of the intestine. The number of genes showing over 2-fold differential gene expression observed under either low-fat or high-fat dietary conditions are presented in the figure. (**B**) A comparison of all genes showing differential expression in response aging in at least one of on the 4 segments of the intestine shows an overlap between the response under low-fat and high-fat conditions. (**C**) By comparing all aging-responsive genes under both low-fat and high-fat conditions a marked difference between the proximal, middle and distal part of the small intestine was observed. (**D**) In total, 124 genes were over 2-fold regulated in the small intestine in response to aging and 104 genes showed a differential aging response in the colon, 27 of these genes were differentially expressed in both segments.

To evaluate the molecular effects of aging, microarray analysis was carried out on RNA isolated from scrapings derived of the proximal, middle and distal part of the small intestine and of the colon after two weeks of dietary intervention. A fold change threshold (2.0 fold) was applied for the identification of differentially expressed genes to determine the most prominent changes in gene expression. The results presented in Figure 2A show that in mice fed a low-fat diet, aging altered expression of only a limited number of genes in the small intestine. However, aging changed expression of a much larger number of genes in the colon, under both low-fat and high-fat conditions. In mice fed a high-fat diet the distal part of the small intestine showed a relative pronounced aging response, but in the proximal and middle segment gene expression between young and old mice turned out to be highly similar. Remarkably, while in the colon aging predominantly induced gene expression, in the small intestine aging mainly suppressed gene expression.

By comparing the total number of differentially expressed genes by aging under low-fat and high-fat conditions in all segments of the intestine together we found that most of the genes that were over 2-fold regulated in mice fed a low-fat diet were also regulated in mice fed a high-fat diet (Figure 2B). Interestingly, a comparison of the 3 segments of the small intestine revealed that only 2 genes were over 2-fold regulated in all segments under either low-fat or high-fat conditions (Figure 2C). Furthermore, 27 genes were differentially expressed in the colon as well as in one or more segments of the small intestine in response to aging (Figure 2D). However, of these 27 genes 15 were oppositely regulated (see Additional file 1: Table S1).

### Increased expression of genes involved in immune response and inflammation in the colon of aged mice

**Figure 3:**
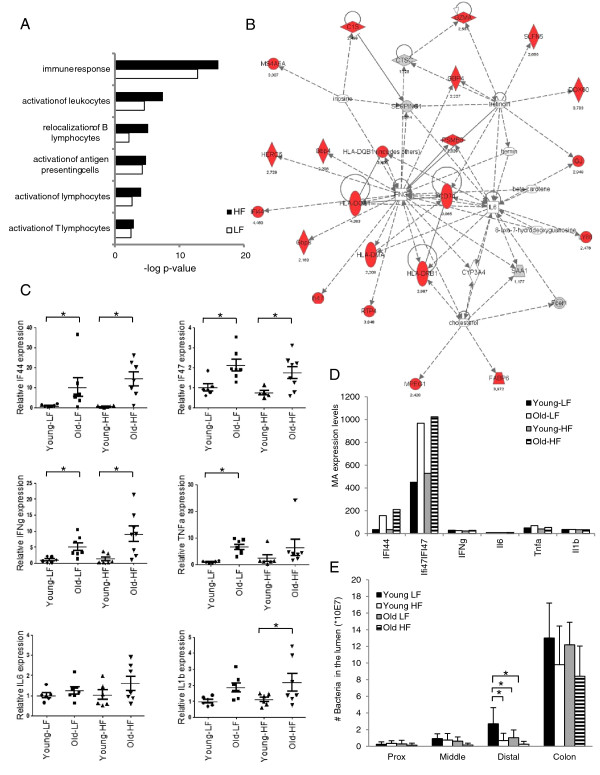
**Enhanced expression of genes involved in immune response and inflammation in the colon of aging mice.** (**A**) Ingenuity Pathway Analysis was carried out on the genes showing at least 2-fold differential expression in the colon of aging mice. Ingenuity Pathway Analysis identified Immune response as the major regulated category. Functions within this category are shown that were significant with a p-value of at least 0.005 (−log p-value 2.3) and at least five genes differentially expressed between old and young mice in the LF group. (**B**) Although INFγ expression was below the threshold level of our microarray data a large number of INFγ-responsive genes showed over 2-fold up-regulation in aging mice. (**C**) Q-PCR analysis confirmed the up-regulation of the INFγ target genes IF44 and IF47 and showed in addition up-regulated expression of INFγ, TNFα and IL-1β but not of IL6 in 21-month-old mice. (**D**) Basal expression levels of various genes measured by microarray analysis and (**E**) total number of bacteria present in the lumen of the 4 segments of the intestine measured by 16S rRNA Q-PCR in old and young mice under low-fat and high-fat conditions. The mean values ± SE were presented, *p < 0.05.

Ingenuity Pathway Analysis (IPA) was applied on the genes showing aging-induced differential expression in the colon to obtain insight into which pathways and networks these genes contribute to. As can be seen in Figure 3A, the group of genes showing enhanced expression in the colon of 21-month-old mice was dominated by genes involved in immune response and inflammation. To compare the differential response of this functional category between the small intestine and the colon in more detail, we selected all genes involved in immune response and inflammation (based on gene ontology) showing over 2-fold differential expression in old mice compared to young mice in at least one of the four segments of the intestine. The results presented in Additional file 2: Table S2 show a large number of genes with increased expression under both low-fat and high-fat conditions specifically in the colon of aging mice. Additional file 2: Table S2 and the IPA network presented in Figure 3B shows that a large number of IFNγ-responsive genes are present amongst the genes displaying enhanced expression in the colon of aging mice. However, since basal gene-expression levels of IFNγ itself were below the threshold of our microarray analysis in all isolated samples, this gene was not included in the analysis. Q-PCR analysis revealed that expression levels of IFNγ in the colon were indeed extremely low, but that significantly enhanced expression in response to aging could be observed (Figure 3C). This expression pattern is similar to that of two IFNγ responsive genes (IF44 and IF47) that showed over 2- fold induction in the colon of old mice (Figure 3C, D). Low grade chronic inflammation is a common phenomenon during aging [29]. By applying Q-PCR analysis on colon samples of the 4- and 21-month-old mice we found that, in line with our microarray results, basal expression of three well-established pro-inflammatory cytokines, TNFα, IL1β and IL6 was very low in both young and old mice (Figure 3C, D). Enhanced expression of TNFα and IL1β was observed in the colon of aging mice while only minor and insignificant effects were found for IL6. Taken together it can be concluded that although basal expression of various pro-inflammatory cytokines is very low, a significant increase could be observed in the colon of aging mice.

To determine whether the observed changes in expression of genes involved in immune response and inflammation were due to alterations in the microflora, 16S rRNA Q-PCR analysis was carried out to analyze the total number of bacteria present in the luminal content of the three segments of the small intestine and of the colon. The results presented in Figure 3E show that, as expected, by far the largest number of bacteria was found in the colon, but no significant changes in total bacterial content between young and old mice were observed. In the distal part of the small intestine a decline in the number of bacteria was found in old mice, but this effect was only significant in mice fed a low-fat diet. In addition, relative abundance of Bacteriodetes and Firmicutes was measured in the colon to determine changes in composition of microbiota. However, no aging-induced changes were observed for these dominant phyla (data not shown).

Together these results indicate that the group of genes showing enhanced expression in the colon of aging mice is dominated by genes involved in immune response and inflammation, but this effect cannot be explained by changes in the total number of bacteria present in the lumen or changes in the Bacteriodetes/Firmicutes ratio.

### Decreased expression of Cubilin in the intestine of aging mice

**Figure 4:**
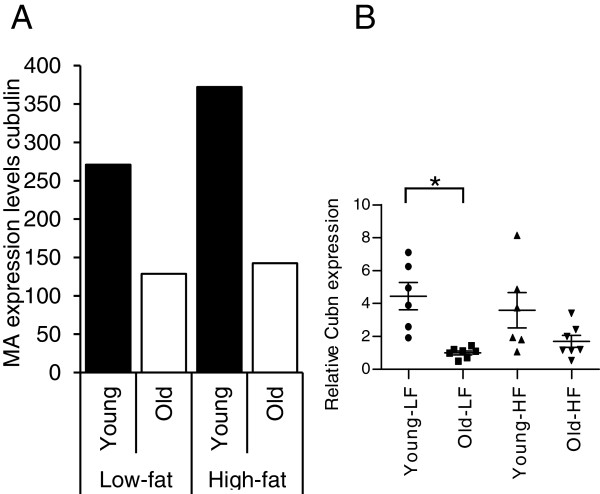
**Effect of aging on Cubn expression in the colon.** (**A**) Microarray analysis and (**B**) Q-PCR analysis revealed decreased expression of Cubn in the colon of aging mice that have received either a low-fat or high-fat diet.

The microarray data presented in Figure 2A show that only a limited number of genes are differentially expressed in the small intestine of aging mice. In line with the observation that energy absorption between young and old mice IPA analyses of the microarray data did not reveal aging-induced changes in common metabolic pathways of macronutrients in the small intestine. Since micronutrient deficiency is common in the elderly we searched for differentially expressed genes involved in micronutrient metabolism. Our microarray data set revealed several genes involved in micronutrient metabolism showing differential expression in the intestine of aging mice. In 21-month-old mice over 2-fold reduction in the expression of Cubilin (Cubn), a gene that mediates the transfer of intrinsic factor-vitamin B12 complex across the epithelial membrane, was found in the colon (Figure 4A). These results could be validated by Q-PCR analysis (Figure 4B). Also in the proximal and middle part of the small intestine of old mice decreased Cubn expression was observed, although these effects were less pronounced than in the colon (data not shown). In addition, basal expression of cytochrome b reductase 1 (Cybrd1), a gene involved in iron metabolism, and metallothionein 2 (MT2) involved in zinc and copper metabolism, showed over 2-fold higher expression in the small intestine of aging mice. However, since expression of both genes is increased and no concomitant differential expression of transporters involved in absorption of these minerals was found it is currently not clear whether these effects contribute to altered micronutrient metabolism in the intestine of aging mice.

In summary, while no major effects on macronutrient metabolism were found in the intestine of old mice, our microarray data revealed that micronutrient metabolism might be affected by aging due to changes in gene expression in the intestine in aging mice. However, further research is required to determine the functional consequences of these changes.

### A high-fat dietary challenge affects gene expression in the small intestine of young and old mice

**Figure 5:**
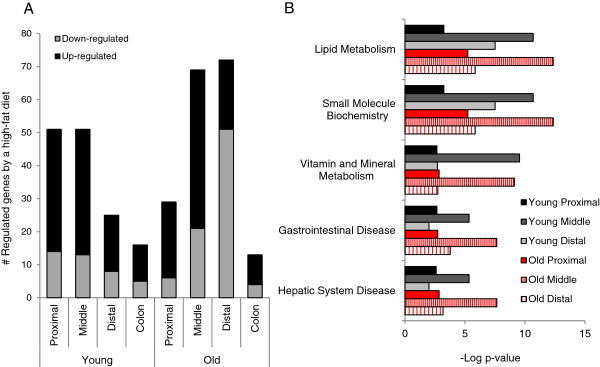
**Differential gene expression induced by a high-fat diet in the 4 segments of the intestine.** (**A**) The number of genes showing at least 2-fold up or down-regulation in the proximal, middle or distal part of the small intestine or in the colon in 4 and 21-month-old mice. (**B**) IPA analysis revealed the major functional categories to which the differentially expressed genes in the small intestine belong.

In addition to the changes in basal gene expression between old and young mice, the microarray data were analyzed to compare the ability of the intestine to respond to a high-fat dietary challenge between the two age groups. As can be seen in Figure 5A, two weeks exposure to a high-fat diet showed, as expected, only a limited effect on gene expression in the colon of both young and old mice. A much stronger response to the high-fat diet was found in the small intestine where also a differential response between old and young mice could be observed. While in young mice the response towards a high-fat diet declined in subsequent sections of the small intestine, in old mice the effect became stronger in the more distal located parts. An overview of all genes showing over 2-fold change in expression in at least one of the 4 segments of the intestine in response to a high-fat dietary challenge is presented in Additional file 3: Table S3.

IPA analysis applied to the genes regulated in response to a high-fat diet in the small intestine of young and old mice indicated that, although the number of genes showing over 2-fold regulation differed between young and old mice, the same pathways were affected in both age groups (Figure 5B and Additional file 4: Table S4). The most pronounced difference in the fat response between old and young mice was detected in the distal part of the small intestine. Analysis of the microarray data revealed that in this segment of the small intestine a large number of genes involved in immune response and inflammatory processes were down-regulated in response to a high-fat diet in old mice (see Additional file 3: Table S3).

Taken together these results imply that although the number of genes over 2-fold regulated by a high-fat dietary challenge is different between young and old mice, the same pathways were affected in the two age groups. This suggests that the intestine of the old mice seems still highly capable in handling a high-fat dietary challenge.

### Decreased global DNA methylation in the intestine of aging mice

**Figure 6:**
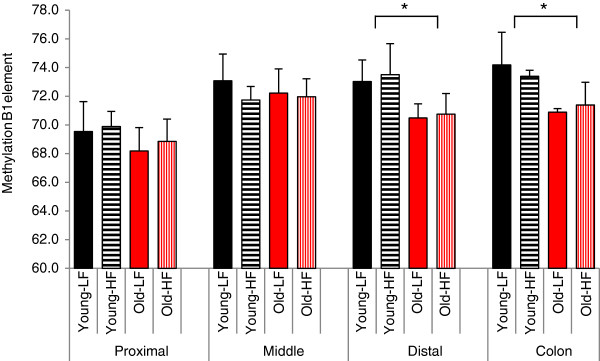
**Global DNA methylation in the 4 segments of the intestine.** Pyrosequencing of B1 elements in the proximal, middle and distal part of the small intestine and in the colon revealed significant global hypomethylation in the distal part of the small intestine and colon of aging mice. Mean values ± SE were presented, *p < 0.05.

Pyrosequencing of B1 elements, a CpG rich repetitive sequence present in the mouse genome, was carried out in DNAs isolated from the same scrapings as used for microarray analysis. As can be seen in Figure 6 a significant decrease in global DNA methylation was found in the distal part of the small intestine and the colon under both low-fat and high-fat conditions in the 21-month-old mice. Also in the proximal part of the small intestine, where the methylation status of the B1 element was found to be lower than in the other segments of the intestine, decreased methylation of the B1 element was found in 21-month-old mice, athough this effect was not significant. No change in global DNA methylation was found in the middle part of the small intestine. Furthermore, the results presented in Figure 6 show that two weeks exposure to a high-fat diet did not induce significant changes in global DNA methylation in young as well as in old mice in all 4 sections of the intestine.

## Discussion

The importance of appropriate functioning of the gut for the maintenance of health has been generally accepted. However, to what extent aging-induced functional decline in the small intestine and colon have a causal effect on the impaired health status of the increasing number of elderly in our modern Western society is still an unresolved issue. A large number of studies have already zoomed in on different features of the aging intestine separately, but in this study we have examined functional, structural and molecular effects of aging in the mouse small and large intestine. The adult life in C57BL/6J mice is characterized by different phases. The young adult phase starts at 3 and ends at 6 months of age and reflects a human age ranging from 20 to 30 years. Mice are considered to be in their old phase when they are between 18 and 24 months of age representing a human age ranging from 56 until 69 years [30,31]. By comparing energy intake between C57BL/6J mice at the age of 4- and 21-months, we found no reduction in either the efficiency of energy absorption of the control 10E% fat diet or of the 45E% high-fat diet. The fact that energy intake is not altered in the mice implies that capacity to digest and absorb macronutrients by the gastrointestinal tract does not decline with age. In line with this observation, our microarray data discloses no significantly altered pathways in macronutrient metabolism in aging mice up to the age of 21-months. A previous study has analyzed gene expression regulation in the duodenum and colon of aging rats [32]. Similar to our findings this study also observed a higher number of differentially expressed genes in the colon compared to the small intestine. Furthermore, in agreement with our results they found that in the colon, aging caused mainly increased gene expression, while in the small intestine most of the differentially expressed genes were decreased. However, while in our study the number of altered genes involved in metabolic processes was very low, Lee and colleagues reported altered expression of genes involved in nutrient digestion and absorption and energy metabolism, although this observation was not further supported by additional functional analyses [32]. Differences between aging in rats and mice, different segments of the intestine analyzed or the use of scrapings versus whole intestine are factors that might contribute to the discrepancy between the obtained results.

Although we did not observe marked changes in expression of genes involved in macronutrient metabolism, changes in expression levels of genes playing a role in micronutrient metabolism including Cubn were detected. Cubn is of crucial importance for the absorption of vitamin B12 by mediating the transfer of the intrinsic factor-vitamin B12 complex over the apical membrane of enterocytes [33]. Our microarray data show decreased expression of Cubn in the proximal and middle part of the small intestine as well as in the colon of aging mice. Vitamin B12 deficiency occurs frequently in elderly humans [34,35] and has been shown to play a causal role in a variety of diseases like depression and impaired cognition [36,37], cardiovascular diseases [38] and osteoporosis [39,40]. Vitamin B12 deficiency in the elderly is mainly due to reduced food intake and, predominantly, malabsorption. It is believed that insufficient release of vitamin B12 from food and impaired coupling to intrinsic factor (IF) due to chronic atrophic gastritis [35] represent the underlying mechanisms of the malabsorption. However, our results imply that reduced expression of Cubn might contribute to the impaired absorption of vitamin B12 in the intestine and that this decreased expression puts the elderly at risk of becoming vitamin B12 deficient.

While aging-induced molecular responses occurred predominantly in the colon, in contrast, the major changes in gene expression induced by a high-fat dietary challenge were found in the small intestine. Furthermore, while IPA analysis of the differentially expressed genes showed that aging did not cause major changes in metabolic pathways, genes involved in a variety of metabolic processes dominated the molecular response induced by the high-fat diet, confirming the results of our previous study [3]. Interestingly, exposure to a high-fat diet for two weeks resulted in increased villi length in the distal part of the small intestine in young and old mice. One could speculate that the capacity of the proximal and middle part of the small intestine is insufficient to clear the intestinal content of all lipids and/or fatty acids in case of a diet containing 45E% of fat. As a consequence, the final part of the small intestine is exposed to increased amounts of fat affecting the tissue structure in this part of the small intestine. The fact that the dietary composition affects tissue morphology of the intestine implies that identical nutritional conditions are of crucial importance to analyze aging-induced changes in tissue morphology of the intestine. In line with some other studies we did not observe aging-induced morphological changes in any of the 4 segments of the intestine while other studies reported changes in tissue morphology of the intestine as a consequence of aging. The inconsistency in the published data might be explained by differences in species used in the different studies or the different age groups that were compared. However, part of the diversity in effects might also be caused by artifacts induced by differences in the dietary conditions [13,41-44].

The increased expression of genes involved in immune response and inflammation observed in the colon of the 21-month-old mice points to an affected immune system in this part of the intestine of aging mice. This observation is in agreement with the fact that changes in the immune system are one of the hallmarks of the aging body. Immunosenescence is the functional decline of the adaptive immune system brought on by natural aging whereby protection against infection by pathogens and the effectiveness of vaccination decline [45,46]. The second aging-induced change in the immune system is called inflammaging which is characterized by a low-grade chronic inflammation process that contributes to the pathogenesis of many age-related diseases [47-49]. A large variety of cells with a defense function are present especially in the lamina propria and the submucosa of the intestine accomplishing immune protection via the innate as well as by the adaptive immune response. Interestingly, our microarray and Q-PCR data clearly show that activity of both branches of the immune system is enhanced in response to aging exclusively in the colon but not in the small intestine of old mice. Expression levels of well-established pro-inflammatory cytokines like IFNγ, TNFα, IL6 and IL1β turned out to be extremely low in the colon of both old and young mice and below the threshold of our microarray analysis. These low expression levels are probably due to the fact that these cytokines are predominantly produced by immune cells in the mucosa which is a rather low percentage of cells in relation to all cells present in the intestinal tissue. Q-PCR analysis confirmed the very low basal expression levels of these pro-inflammatory cytokines, yet a weak but significant induction of IFNγ TNFα and IL-1β in the colon of aging mice was observed. This result suggests that low-grade inflammation might be present in the colon of the aging mice in our study, although it should be noted that no altered expression of a number of established inflammation markers like Toll-like receptors (TLRs), C-type lectin receptors (CLRs) and retinoic acid-inducible receptors (RLRs) [50] was detectable.

Changes in the gut microbiota in terms of composition and functionality during the process of aging have previously been reported [19,20,51] and it has been postulated that these changes might contribute to the development of immunosenescence and inflammaging [18,52]. To establish whether the enhanced expression of genes playing a role in the immune system are due to modifications in the microbiota we measured the total number of all bacteria and of the two most prominent phyla colonizing the colon, Bacteriodetes and Firmicutes, in the luminal content of the colon. We did not observe aging-related changes. More advanced techniques like pyrosequencing are required to determine whether total number of bacteria and changes in the composition of the microbiota might play a causal role in the observed changed expression of immune-related genes in the colon of our aging mice. Although it is difficult to assess the physiological consequences of the enhanced expression of genes involved in inflammation and immune response, it seems most likely that this effect is important for the health status of the aging colon.

Interestingly, also colorectal cancer, which occurs with increased frequency in the elderly, has been related to an altered immune status of the colon [53-55]. The fact that we only found increased expression of immune-related genes in the colon, but not in the small intestine is intriguing since tumor formation is a very rare event in the small intestine and molecular evidence explaining the extreme difference in incidence of tumor formation in the two segments of the intestine is currently still lacking. In contrast to what has previously been found in the colon of aging rats [22], none of the well-established genetic markers of colorectal cancer (i.e. APC, KRAS, p53 etc.) or genes involved in cell cycle arrest and apoptosis [56] showed marked differential expression in the colon of the old mice in our study. This observation might be related to the fact that colorectal cancer is only a rare spontaneous event in C57BL/6J mice and that specific predisposing mutations [57] or carcinogenic agents [58,59] are required to induce colorectal cancer in this mouse strain. Apart from genetic events epigenetic modifications have become important markers of tumor development, in particular of colorectal tumors [60]. Hypomethylation of repetitive sequences is frequently observed in tumor tissue, but is also recognized to be an established feature of normal aging tissue [60]. Interestingly, our results show differential demethylation between the various segments of the intestine of normal old mice. Since no significant demethylation in the proximal part of the small intestine is observed, it can be speculated that this part of the intestine is less prone to tumor development. Further research is required to prove this hypothesis and to establish whether changes in DNA methylation are responsible for the altered levels of gene expression observed in the colon of our aging mice in line with what previously has been reported [21].

## Conclusion

The results obtained in this study did not reveal aging-induced morphological changes in neither the small intestine nor the colon of 21-month-old mice. Furthermore, no decrease in macronutrient metabolic functioning of the intestine was detectable. In agreement with these observations, the results obtained by microarray analysis indicated that macronutrient metabolism seems to function appropriately in old mice. In contrast however, gene expression patterns reveal that micronutrient metabolism might be affected in old mice which might contribute to some of the micronutrient deficiencies frequently occurring in the aging segment of our society. Furthermore, it can be speculated that the decrease in global DNA methylation and the altered status of the immune system of the colon might play a causal role in the decreased health status of this organ at old age.

## Competing interests

The authors declare that they have no competing interests.

## Authors’ contributions

WS, NW, NIJ, EK, LG and MM participated in the design and/or supervision of the study. CL, SK and MG performed the experimental work such as sample preparation, Q-PCR, Global DNA methylation, histological analysis. MG hybridized the microarrays. MB assessed the quality control of the microarrays and performed the IAP analysis. WS and MB performed microarray analysis and drafted the manuscript. NW, MB, NIJ, EK, LG and MM provided valuable feedback on the initial draft. All authors read and approved the final manuscript.

## Pre-publication history

The pre-publication history for this paper can be accessed here:

http://www.biomedcentral.com/1755-8794/5/38/prepub

## Supplementary Material

Additional file 1 **Table S1.** All genes differential expressed in one or more segments of the intestine in old mice fed a low-fat or high-fat diet. Click here for file

Additional file 2 **Table S2.** Immune response and inflammatory genes regulated in old mice fed a low-fat or high-fat diet. Click here for file

Additional file 3 **Table S3.** All genes differential expressed in one or more segments of the intestine in response to a high-fat diet. Click here for file

Additional file 4 **Table S4.** Genes present in the IAP functional categories presented in Figure 5B regulated by a high-fat diet in the distal part of the small intestine in young and old mice. Click here for file
